# Micro-ureteroscopy for treatment of pelvic ureteral stone in pediatric patient

**DOI:** 10.1590/S1677-5538.IBJU.2018.0223

**Published:** 2019-07-27

**Authors:** Diogo Nunes-Carneiro, João Ferreira Cabral, Avelino Fraga, Vítor Cavadas

**Affiliations:** 1 Departamento de Urologia de Centro Hospitalar do Porto, Instituto de Ciências Biomédicas de Abel Salazar, Porto, Portugal

## Abstract

**Introduction:**

During the last years there has been an effort in miniaturizing the endoscopic devices.

The video presents an alternative for the management of distal ureteral stone, using a ureteral access of 4.85 Fr and 27 cm of length, previously described as micro-ureteroscopy.

**Material and Methods:**

This procedure was performed through a 3-part all-seeing needle, consisting of micro-optics 0.9 mm in diameter with a 120-degree angle of view, an irrigation channel and an integrated light.

**Clinical Case:**

Seven year-old boy, with history of preterm birth (29 weeks) was referred to our consultation complaining of left back pain and an elevation of serum creatinine.

The renal ultrasound revealed a left ureterohydronephrosis, caused by a 10 mm stone located 13 mm from the ureterovesical junction.

The patient underwent a micro-ureteroscopy with laser lithotripsy. The stone was fragmented with an average energy of 0.5 J with 12 Hz of frequency. The total energy spent was 12514 J. At the end of the procedure, a double J stent was placed.

The procedure lasted 45 minutes and was uneventful. The patient was discharged 24h after the procedure without complaints and remained stone free.

**Conclusion:**

Micro-ureteroscopy is a safe and effective technique in distal ureteral lithiasis treatment in children. The small dimensions of the equipment increase the safety of the procedure making this a good option for the treatment of ureteral stones in children.

## ARTICLE INFO

**Available at: http://www.intbrazjurol.com.br/video-section/20180223_Nunes-Carneiro_et_al**

**Int Braz J Urol. 2018; 44 (Video #9): 639-639**

